# The Prevalence of Psychiatric Distress and Associated Risk Factors among College Students Using GHQ-28 Questionnaire

**Published:** 2017-07

**Authors:** Jalal POOROLAJAL, Ali GHALEIHA, Nahid DARVISHI, Shahla DARYAEI, Soheila PANAHI

**Affiliations:** 1. Dept. of Epidemiology, School of Public Health, Hamadan University of Medical Sciences, Hamadan, Iran; 2. Research Center for Health Sciences, Hamadan University of Medical Sciences, Hamadan, Iran; 3. Dept. of Psychiatry, School of Medicine, Hamadan University of Medical Sciences, Hamadan, Iran; 4. Counseling Center, Hamadan University of Medical Sciences, Hamadan, Iran

**Keywords:** Mental disorders, Substance-related disorders, Suicide, Smoking, Unsafe sex

## Abstract

**Background:**

Adolescent and young adults are at increased risk of psychiatric distress and serious disability. We estimated the prevalence and associated risk factors of psychiatric distress among the college students of Hamadan University of Medical Sciences, Iran.

**Methods:**

We performed this cross-sectional study, from Jan to May 2016 at Hamadan University of Medical Sciences, Hamadan, Iran. Students filled out voluntarily an anonymous self-administered questionnaire, including demographic characteristics, personal information, behavioral risk factors, and a validated Persian version of the GHQ-28 questionnaire, including somatic symptoms (items 1–7), anxiety/insomnia (items 8–14), social dysfunctions (items 15–21), and severe depression (items 22–28).

**Results:**

Of 1259 participants, 518 (41.1%) had psychiatric distress, 166 (13.2%) had heterosexual intercourse, 100 (8.0%) had homosexual intercourse, 204 (16.2%) were smokers (31.6% in males and 6.3% in females), 124 (9.9%) reported a history of using opium/psychedelic substances, 204 (16.2%) reported suicide thought, and 103 (8.2%) had attempted suicide at least once in the past. After adjusting odds ratio (95% CI) for age and sex, psychiatric distress were significantly associated with emotional breakdown 2.67 (2.09, 3.40), heterosexual intercourse 2.56 (1.82, 3.62), homosexual intercourse 2.42 (1.57, 3.71), smoking 3.19 (2.29, 4.45), substance abuse 5.03 (3.26, 7.76), suicide thought 7.81 (5.42, 11.27), suicide attempt 5.64 (3.49, 9.12), uninterested in the discipline 2.29 (1.70, 3.07), and non-optimistic about future 2.16 (1.63, 2.86).

**Conclusion:**

A majority of college students had psychiatric distress and a substantial number of them reported one or more high-risk behaviors that if neglected, may severely impair the students’ function and influence their subsequent development and productive lives.

## Introduction

The burden of mental health problems continues to grow with considerable impacts on health economic consequences worldwide (1). About 10% to 20% of children and young adults experience mental illnesses. About half of all mental disorders begin by the age of 14 and three-quarters by mid-20s (2). Mental disorders are recognized with different presentations, including distorted thoughts, altered perceptions, impaired emotions, abnormal behavior, and atypical communication (1).

College students are a large part of young adults in whom the prevalence of certain mental disorders has been reported to be relatively high (3–5). Although college can be an exciting time for most youth, it can also be often associated with considerable pressures that may exacerbate the risk of mental illness such as long hours of study, irregular sleep patterns, academic pressures, financial pressures, and living away from home for the first time (3, 4, 6, 7). Depression, anxiety disorders, eating disorders, and substance use disorders are common mental health problems among college students (4, 8, 9).

Adolescents and young adults with mental disorders are at increased risk of serious disability in all regions of the world. If untreated, these disorders may severely impair adolescents’ functioning at college and influence their subsequent development (2, 8). The causes of mental disorders are usually complex and are dependent on the individual and the social environment. Despite the impact of mental disorders on the general health, the risk factors of such disorders among college students have not been properly investigated in developing countries. Some important risk factors such as high-risk sexual behaviors, substance abuse, suicidal behaviors, particularly among youth, are not well investigated and are not clearly reported in Iran because of cultural and religious limitations while these risk factors exist, although they are not evaluated or even they are not reported. Ignoring this risk factor will not solve the problem. Addiction to social networks and problematic internet use are new risk factors among the today’s youth. These are undeniable problems of our society believed and must be investigated until we find a remedy for them. Until reliable information of the burden and causes of mental disorders is collected, it is difficult or even impossible to design effective intervention strategies and to carry out preventive measures.

The present study was conducted in order to portray the prevalence and associated risk factors of psychiatric distress among college students.

## Methods

The Ethics Committee of the Hamadan University of Medical Sciences approved the study. We invited the college students of the Hamadan University of Medical Sciences, Hamadan, Iran, to participate in this cross-sectional study from Jan to May 2016.

According to the results of an epidemiological study, the prevalence of depression, anxiety, eating disorders, and harmful drinking among the Australian students was reported 8%, 13%, 14%, and 8%, respectively (3). Because of these results, assuming *P* to be 0.08, we arrived at a sample size of 1105 at 95% significance level. Considering the refusal rate, we increased the sample size by about 20% and arrived at a maximum of 1338. In order to increase the generalizability of the results, we performed a proportional random sampling. For this purpose, we divided Hamadan University of Medical Sciences into different colleges (strata) and then, we took random samples of each stratum. We just enrolled college students from all disciplines passed at least one semester and excluded the newcomers.

The participants filled out voluntarily an anonymous self-administered questionnaire. The data collection tool consisted of two parts. The first part of the questionnaire included demographic characteristics, personal information, and behavioral risk factors. The second part of the questionnaire was the Persian version of the GHQ-28 questionnaire. The GHQ-28 was developed and introduced as a screening tool to detect those likely to have or to be at risk of developing psychiatric disorders. The GHQ-28 is a 28-item measure of the common mental health problems, including somatic symptoms (items 1–7), anxiety/insomnia (items 8–14), social dysfunctions (items 15–21), and severe depression (items 22–28) (10). This questionnaire has been translated into 38 languages (11). The Persian version of the questionnaire was developed (12). They purposed a cutoff point of 23. Based on the Likert scoring method, the sensitivity and specificity of the questionnaire were estimated 70.5% and 92.3%, respectively. The reliability of the questionnaire based on the value of Cronbach’s alpha was 87%.

We used the chi-squared test for analysis of categorical variables. We also used the simple and multiple logistic regression analysis to measure the association between psychiatric distress and the behavioral risk factors. All statistical analyses were performed at a significance level of 0.05, using Stata software, ver. 11 (StataCorp, College Station, TX, USA).

## Results

We identified 1338 eligible college students, 45 subjects refused to fill out questionnaires, 30 did not return questionnaires, and 4 were excluded from the analysis because they did not complete questionnaires. The analysis was based on data from the remaining 1259 participants.

The acceptance rate was 94%. The mean (SD) age of the participants was 22.54 (3.34) with a range of 18 to 49 yr. Of 1259 participants, 741 (58.9%) had no distress and 518 (41.1%) had psychiatric distress. The details of psychiatric disorders, including somatic symptoms, anxiety or insomnia, social dysfunctions, and severe depression are given in Table 1.

**Table 1:** Prevalence of psychological distress among study population

**Psychological distress**	**Without distress**		**With distress**	
	**n**	**%**	**n**	**%**
Somatic symptoms (Items 1–7)	793	63.0	466	37.0
Anxiety/Insomnia (Items 8–14)	787	62.5	472	37.5
Social dysfunctions (Items 15–21)	529	42.0	730	58.0
Severe depression (Items 22–28)	956	75.9	303	24.1
Total (Items 1–28)	741	58.9	518	41.1

The demographic and personal characteristics of the study population are given in Table 2. The prevalence of psychiatric distress was higher among students aged 26 to 29 yr (52.7%; *P*=0.027). Those who were males (46.4%; *P*=0.003), fourth birth order (49.2%; *P*=0.092), divorced (48.8%; *P*=0.547), lived in surrounding towns (45.4%; *P*=0.053), lived with parents (40.8%; *P*=0.887), with the educational level of MSc (65.6%; *P*=0.001), students of the college of pharmacology (55.4%; *P*=0.070), with years of education ≥7 yr (66.0%; *P*=0.007), uninterested in the discipline (58.5%; *P*=0.001), and non-optimistic about future (57.1%; *P*=0.001).

**Table 2:** Comparison of the demographic and personal characteristics of the college students with and without psychiatric distress based on GHQ-28 questionnaire using chi-squared test

**Variables**	**Without distress (n=741)**		**With distress (n=518)**		**Total**	***P* value**
	**n**	**%**	**n**	**%**		
Sex						0.003
Male	265	53.6	229	46.4	494	
Female	476	62.2	289	37.8	765	
Age group (yr)						0.027
18–21	344	62.2	209	37.8	553	
22–25	317	57.6	233	42.4	550	
26–29	53	47.3	59	52.7	112	
≥30	27	61.4	17	38.6	44	
Birth order						0.092
First	270	60.7	175	39.3	445	
Second	242	61.0	155	39.0	397	
Third	133	58.3	95	41.7	228	
Forth	96	50.8	93	49.2	189	
Marital status						0.547
Single	620	58.9	432	41.1	1052	
Married	99	60.4	65	39.6	164	
Divorced	22	51.2	21	48.8	43	
City						0.053
Hometown	241	63.1	141	36.9	382	
Surrounding towns	216	54.6	180	45.4	396	
Towns of other provinces	284	59.0	197	41.0	481	
Residence						0.877
Dormitory	522	58.7	367	41.3	889	
Parents’ house	219	59.2	151	40.8	370	
Educational level						0.001
BSc	374	62.4	225	37.6	599	
MSc	33	34.4	63	65.6	96	
MD	316	60.8	204	39.2	520	
PhD	18	40.9	26	59.1	44	
College						0.070
Medicine	233	63.7	133	36.3	366	
Dentistry	61	59.2	42	40.8	103	
Public health	140	57.1	105	42.9	245	
Paramedical	141	56.6	108	43.4	249	
Pharmacology	37	44.6	46	55.4	83	
Nursing/Midwifery	97	59.9	65	40.1	162	
Rehabilitation	32	62.8	19	37.2	51	
Years of education						0.007
1–2	139	54.5	116	45.5	255	
3–4	238	57.5	176	42.5	414	
5–6	76	48.4	81	51.6	157	
≥7	17	34.0	33	66.0	50	
Interest in the discipline						0.001
Yes	646	62.7	384	37.3	1030	
No	95	41.5	134	58.5	229	
Optimistic about future						0.001
Yes	629	63.0	369	37.0	998	
No	112	42.9	149	57.1	261	

The association between psychiatric distress and some high-risk behaviors is given in Table 3. Of 1259 college students, 166 (13.2%) had heterosexual intercourse, 100 (8.0%) had homosexual intercourse, 204 (16.2%) were smokers, including 156 males (31.6%) and 48 females (6.3%), 124 (9.9%) reported a history of using opium or psychedelic substances, 204 (16.2%) reported suicide thought, and 103 (8.2%) had attempted suicide at least once in the past. The mean (SD) use of social networks was 4.68 (3.43) h/day ranged from 0.5 to 20.

**Table 3:** Association between psychological distresses based on GHQ-28 questionnaire and high-risk behaviors

**Variables**	**Without istress (n=741)**	**With distress (n=518)**	**Unadjusted OR (95% CI)**	***P* value**	**Adjusted OR (95% CI)^a^**	***P*-value**
Having emotional breakdown						
No	562	277	1.00		1.00	
Yes	179	241	2.73 (2.15, 3.48)	0.001	2.67 (2.09, 3.40)	0.001
Having heterosexual intercourse						
No	676	412	1.00		1.00	
Yes	62	104	2.75 (1.96, 3.86)	0.001	2.56 (1.82, 3.62)	0.001
Having homosexual intercourse						
No	703	453	1.00		1.00	
Yes	37	63	2.64 (1.73, 4.03)	0.001	2.42 (1.57, 3.71)	0.001
Current cigarette smoker						
No	670	383	1.00		1.00	
Yes	71	133	3.28 (2.39, 4.49)	0.001	3.19 (2.29, 4.45)	0.001
Current substance abuser						
No	711	424	1.00		1.00	
Yes	30	94	5.25 (3.42, 8.06)	0.001	5.03 (3.26, 7.76)	0.001
Having suicide thought						
No	700	355	1.00		1.00	
Yes	41	163	7.84 (5.44, 11.30)	0.001	7.81 (5.42, 11.27)	0.001
History of suicide attempt						
No	718	438	1.00		1.00	
Yes	23	80	5.70 (3.53, 9.20)	0.001	5.64 (3.49, 9.12)	0.001
Using social networks						
No	97	55	1.00		1.00	
Yes	643	459	1.26 (0.89, 1.79)	0.200	1.24 (0.87, 1.77)	0.231
Interest in the discipline						
Interested	646	384	1.00			
Uninterested	95	134	2.37 (1.77, 3.18)	0.001	2.29 (1.70, 3.07)	0.001
Optimistic about future						
Yes	629	369	1.00			
No	112	149	2.27 (1.72, 2.99)	0.001	2.16 (1.63, 2.86)	0.001

a Adjusted for age and sex

As shown in Table 3, after adjusting the odds ratio (95% confidence interval) for age and sex, psychiatric distress was significantly associated with having emotional breakdown 2.67 (2.09, 3.40), heterosexual intercourse 2.56 (1.82, 3.62), homosexual intercourse 2.42 (1.57, 3.71), smoking 3.19 (2.29, 4.45), substance abuse 5.03 (3.26, 7.76), suicide thought 7.81 (5.42, 11.27), suicide attempt 5.64 (3.49, 9.12), uninterested in the discipline 2.29 (1.70, 3.07), and non-optimistic about future 2.16 (1.63, 2.86). Psychiatric distress was not statistically associated with using social networks 1.24 (0.87, 1.77).

## Discussion

Our findings revealed that a majority of college students had psychiatric disorders, including somatic symptoms, anxiety or insomnia, social dys-functions, and severe depression. We also indicated that psychiatric distress was significantly associated with having emotional breakdown, high-risk sexual behaviors, smoking, substance abuse, suicide thought, and suicide attempt. Suicide is a multifactorial phenomenon (13, 14) that is strongly correlated with mental disorders (15, 16). Furthermore, high-risk behaviors such as substance use disorder (17), alcohol use disorder (18), and smoking (19) are associated with an increased risk of suicidal behaviors.

The age-standardized estimated prevalence of current tobacco smoking among Iranian aged 15 yr or more was reported 11.7% (22.4% in males and 1.0% in females) (20). While according to our findings, the prevalence of current smoking among college students was 16.2% (31.6% in males and 6.3% in females). The prevalence of smoking was significantly higher among college students than among the general population. Furthermore, these students were studying in different colleges of a medical university, expected to be well aware of the health consequences of cigarette smoking. If this study had been conducted in non-medical universities, the prevalence of smoking might have been reported much higher. About 80% of youth were experienced their first smoking before age of 15 yr (21). Hookah, cigarette smoking, unsafe sexual behaviors, violent behavior, alcohol intake were the most frequent high-risk behaviors among youth, respective(22). Therefore, prevalence of smoking among adolescent and young adults is a serious warning signal that if neglected, may severely impair the students’ function and influence their subsequent development.

We found a positive association between illegal sexual behavior and psychiatric distress. This is consistent with previous studies of student populations. A study conducted on 6044 university students in Australia indicated that students identified as homosexual or bisexual were three times more likely of having mental disorders, compared with students who were heterosexual (3). In the USA, the relationship between mental health and sexual orientation was investigated among 27454 universities and found that gay, lesbian, bisexual or unsure students were at greater risk of having mental disorders than heterosexual students (23).

The prevalence of psychiatric distress is relatively high among young adults, particularly among college students, as was the case in our study. Anxiety, depression, psychological distress were the most common mental distress among medical students outside North America (4). A web-based cross-sectional survey in Australia has identified variables associated with common mental disorders in 6044 Australian college students. According to the results of this study, depression, anxiety, eating disorders, and harmful drinking were common mental disorders that affected 30% of the students. These disorders were much higher among female sex, age of 25 to 34, subjects with low income, homosexual and bisexual students (3). A survey of 34324 Minnesota college students was conducted based on self-reported mental disorders and distress by sexual orientation. This study showed that the prevalence of mental disorders was significantly higher among lesbian, gay, and bisexual students than heterosexual students (24). The variation in student mental health was assessed across US college students. Of 43210 students were indicated that mental health problems were higher in doctorate-granting institutions, baccalaureate colleges, institutions with small enrollments, and schools with strong residential systems (5).

This study had a few limitations. The questionnaire that we used in this study included a number of sensitive questions. Answers to question about sexual activities rely heavily on self-reported data. People usually do not give correct answer to such questions (25). Considering this issue and the rejection rate of 6%, our results underestimated the high-risk behaviors among the college students. The real prevalence of high-risk behaviors is higher than what we reported in this paper. Furthermore, the results of this cross-sectional study only reported an association between psychiatric disorders and high-risk behaviors. However, the association dos not necessarily imply a cause-and-effect relationship because exposures and outcome were determined at the same time. In such cases, it is often not possible to establish a temporal relationship between the exposure and the onset of outcome (26). Therefore, it not clear whether psychiatric disorders proceeded and caused high-risk behaviors or vice versa.

Despite this limitation, this study provided beneficial information about the burden and potential risk factors of mental disorders. Some of the risk factors reported in this paper are considered taboo in our country and information about them is hardly reported. We investigated and highlighted the prevalence of these risk factors among students that comprise the active part of our community. Part of information reported in this article is shocking and is rarely reported in the previous publication. Such information can be used for planning effective intervention strategies and implementing preventive measures. However, behavioral risk factors are closely related to sociocultural situation. Therefore, the results of this study may not be generalized to settings with different sociocultural situations. Therefore, each setting requires investigating the prevalence and associated risk factors of psychiatric disorders among their own young adults.

## Conclusion

Nearly one-half of university students had psychiatric distress and a majority of them had at least one or more high-risk behaviors. If these risk factors are neglected, may severely impair the students’ performance and influence their subsequent development and productive lives. This study properly revealed the high prevalence of the mental health problem among college students and provided beneficial information of factors associated with the problem. These findings may be useful for policymakers planning effective prevention strategies.

## Ethical considerations

Ethical issues (Including plagiarism, informed consent, misconduct, data fabrication and/or falsification, double publication and/or submission, redundancy, etc.) have been completely observed by the authors.
